# Influence of Bentonite Particles on the Mechanical Properties of Polyester–Sisal Fiber Composites

**DOI:** 10.3390/polym15193963

**Published:** 2023-09-30

**Authors:** José Luis Valin Rivera, Cristian Rodolfo Valenzuela Reyes, Arturo Andrés Quinteros Wachtendorff, Angel Rodríguez Soto, Meylí Valin Fernández, Roberto Iquilio Abarzúa, Alvaro González Ortega, Gilberto García del Pino, Francisco Rolando Valenzuela Diaz

**Affiliations:** 1 Escuela de Ingeniería Mecánica, Pontificia Universidad Católica de Valparaíso, Valparaíso 2340025, Chile; cristian.valenzuela@arctechsolar.com (C.R.V.R.); a.quinterosw@gmail.com (A.A.Q.W.); angel.rodriguez@pucv.cl (A.R.S.); roberto.iquilio@pucv.cl (R.I.A.); alvaro.gonzalez.o@pucv.cl (A.G.O.); 2 Department of Mechanical Engineering (DIM), Faculty of Engineering (FI), University of Concepción, Concepción 4030000, Chile; mvalin@udec.cl; 3 Department of Mechanical Engineering, State University of Amazonas, Manaus 69850-020, Brazil; gpino@uea.edu.br; 4 Department of Materials Engineering and Metallurgy, University of São Paulo, São Paulo 05508-030, Brazil; frrvdiaz@usp.br

**Keywords:** sisal fiber, bentonite, polymer composite, mechanical properties

## Abstract

As a part of the mission to create materials that are more environmentally friendly, we present the following proposal, in which a study of the mechanical properties of composite materials comprising a polyester resin with sisal fiber and bentonite particles was conducted. Sisal fiber was added to a matrix in percentages ranging from 5% to 45% in relation to the polyester resin weight, while bentonite remained fixed at 7% in relation to the polyester resin weight. The specimens were manufactured by compression molding. The mechanical properties were analyzed by tensile, bending, impact, stepped creep, and relaxation tests. In addition, energy-dispersive X-ray spectroscopy and scanning electron microscopy analyses were carried out to analyze the composition and heterogeneity of the structure of the composite material. The results obtained showed that 7% of bentonite added to the matrix affects the tensile strength. Flexural strength increased by up to 21% in the specimens with a 20% addition of sisal fiber, while the elastic modulus increased by up to 43% in the case of a 20% addition of sisal fiber. The viscoelastic behavior was improved, while the relaxation stress was affected.

## 1. Introduction

Due to the current need to care for the environment and achieve sustainability goals, industries need to create new production technologies to reduce their consumption of raw materials. This can be achieved by improving the properties of materials by applying heat treatments [1] and using renewable materials and composite materials [2,3,4]. Composite materials have widespread uses within different areas of engineering, such as in the aerospace, civil, marine, biomedical, and automotive industries [1,2,3,4,5], with potential to replace conventional materials such as metals and their alloys. Composites reinforced with synthetic fibers, such as glass, carbon, and aramid fibers, are replaced by vegetable fibers, such as jute, sisal, coconut, banana, curauá, henequen, etc. [6,7,8,9,10]. The application of vegetal fibers as reinforcements in composite materials has increased in recent years to replace synthetic fibers due to the characteristics of natural fibers, such as their low cost, low density, high specific modulus, economic and environmental advantages, biodegradability, abundance, and many technical qualities [5,6,7,8].

The mechanical properties of vegetal fibers have been studied by several authors [9,10]. Guerra-Silva et al. [11] determined the resistance of the matrix–fiber interface using a polyester resin matrix and different quantities of henequen fiber, obtaining excellent tensile strength and impact resistance. Gupta and Srivastava [12] analyzed the mechanical properties of an epoxy matrix composite with different quantities of sisal fibers showing that the fiber provides an improvement in tensile (147%), flexion (112%), and impact (288%), when compared with the pure resin. Mancinoa et al. [13] determined the mechanical properties of different composites for structural applications based on biodegradable resin and sisal fiber. They obtained 215 MPa of tensile stress and a Young’s modulus of 15 GPa, concluding that the composites could replace technical materials, such as aluminum alloys and fiberglass composites. Other authors have determined the mechanical properties of different composites for structural applications [14,15,16].

The use of particles together with fibers in hybrid composites is currently being developed by many researchers, such as Rajesh et al. [17], with their study of the mechanical properties of a composite of madar fibers (*Calotropis gigantea*) and clay particles as reinforcement. Their study concluded an improvement of almost 8% in tensile strength, showing 25.16 MPa for a composite consisting of 10% fiber weight and 1% particle weight. Shuvo et al. [18] determined that the composite material in a matrix with 5% jute fiber and 3% bentonite particles as reinforcement presented tensile strength improvements with a value of 21.5 MPa. Similarly, the research carried out by Hasan and Mollik [19] presents results that conclude that an unsaturated polyester compound reinforced with 3% jute fiber by weight and 1% bentonite particles showed improvements in resistance to traction and bending. Their results demonstrated increases from 12.5 to 21.5 MPa and 85.28 to 136 MPa, implying 58% and 63% improvements in traction and bending strength properties, respectively. Other researchers, such as Garcia del Pino [20], have also obtained good results using curauá fibers and organophilic clay in composites with an epoxy resin matrix and in a polyester matrix [21]. Kieling’s work used Tucuma fibers and Kaolin in an epoxy resin matrix [22].

The addition of plant fibers and bentonite microparticles allows the effective global properties of the composites to be modified. With the above, properties such as tensile strength, elastic modulus, and others can increase or influence creep phenomena and stress relaxation. This allows materials to be designed for specific applications, such as packaging [23], construction, and automotive and aircraft components [24,25].

Among the materials mentioned above, sisal fibers are widely used in Chile, mainly made by hand with low production levels. At the same time, bentonite is abundant in natural environments but does not yet have a specific use in this field. Considering the characteristics and properties reported in the literature for both materials, our research group decided to manufacture and study a composite material with the addition of sisal fibers, bentonite particles, and polyester resin, with the latter being chosen due to it being easily accessible and for its mechanical properties. The objective of this work was to perform the mechanical characterization of the composite of a polyester matrix reinforced with sisal fibers and bentonite particles based on mechanical tests, energy-dispersive X-ray spectroscopy (EDS) analysis, and barrier electron microscopy (BEM). The novelty of this work is based on the following:

▪ The literature does not cover the combinations and analyses used here.
▪ This study allows a deeper understanding of the mechanical behavior of this composite material under various working conditions.

## 2. Materials and Methods

### 2.1. Materials

**Figure 1:**
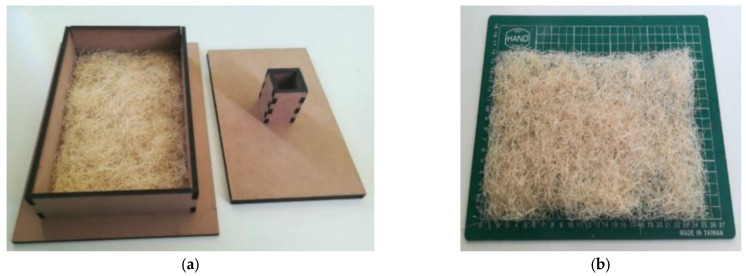
Manual pre-molded of sisal fibers inside an MDF press: (**a**) box for manual pre-molding of fibers; (**b**) pre-molded fiber layer.

The polyester resin POLIPOL 3401-H15 and the methyl ethyl ketone catalyst (Peroxide MEK) were used for the studied composite material fabrication. Figure 1 presents the formation of the crosslinked polyester, while Table 1 presents some of its physical properties. The sisal fibers were obtained from commercial braided sisal separated into strands with an average diameter of 0.25 mm and a cross-sectional area of 0.05 mm^2^ [11]. The bentonite used was Rheotix VP bentonite of the calcium type, supplied by the University of São Paulo (USP).

### 2.2. Composite Fabrication

The sisal fiber was cut to 25.4 mm on a PA-CIFIC PAC01005 guillotine, following the critical length criterion presented by Fernández et al. [19]. Equation (1) presents the expression to determine the critical length *l_c_*, where *σ_f_* is the maximum stress of the fiber, *d_f_* is the fiber’s diameter, and *τ_c_* is the matrix’s shear stress.

(1) $$l_{c}=\frac{\sigma_{f}d_{f}}{2\tau_{c}}$$

The compression molding technique was used to manufacture the plates from which the specimens are obtained to carry out the mechanical characterization tests, following a procedure similar to that presented by Fernández et al. [26]. Once the fibers have been cut, they are weighed on a digital scale with a maximum capacity of 100 g and a minimum division of 0.01 g. Then, the fiber is scattered randomly inside a mold made of medium density fiberboard (MDF), of 5.5 mm of thickness, which was cut using a 1309× laser cutter from the manufacturer BODOR, as presented in Figure 1a,b. This function is to give a preform of the fibers in order to avoid subsequent manipulation of these in the steel mold, which can produce voids in the layers. This process allowed us to obtain the exact measurements of the final plate (156 × 250 mm), as presented in Figure 1. This part of the procedure was not reported by Fernández et al. [26], but is a modification developed for the present work.

The resin and the catalyst were weighed according to the specifications of the manufacturer Masterfibra, using a digital scale with a maximum capacity of 100 g and a minimum division of 0.01 g, by dosing through a peroxide dropper, using a digital scale with a maximum capacity of 3 kg and a minimum division of 1 g for polyester resin. Three 1045 steel molds were used to manufacture the plates. These molds were designed to ensure that the plate measured 250 × 156 × 3 mm.

The bentonite is weighed following the proportion of 7% in relation to the total weight of the resin, which corresponds to 9.45 g per plate. The bentonite was added slowly and in moderate amounts to ensure correct impregnation and a sieve strainer was also used to avoid the formation of lumps.

The mixtures with different proportions of materials were poured on the fibers arranged in the steel molds and were simultaneously placed in the Microcomputer-Controlled Electronic Universal Machine WDW-2006. The universal machine was set at a constant pressure of 42 kN for 24 h. Once this time had elapsed, the molds were removed from the Universal Machine, and the plates were recovered, having cured for 30 days. After 30 days of curing, the specimens were cut for the experimental tests. The designs of the specimens were made under the standards corresponding to each test. The 1309× laser engraving and cutting machine from the manufacturer BODOR was used to cut the specimens, with a cutting speed of 14 mm/s.

### 2.3. EDS and SEM Characterization

The bentonite was characterized through energy-dispersive X-ray spectroscopy (EDS) to obtain its elemental composition. For this purpose, a barrier electron microscope (MEB), model Hitachi SU3500, was used, which was coupled to a Bruker XFlash 410 m X-ray diffraction (DRX) detector. To observe the surface of the fractures generated in the traction probes, a ZEISS scanning electron microscope, model EVO MA 10, was used. For this, samples of dry, untreated, and unmetalled bentonite were used and placed on a support to be later analyzed.

The fractured surfaces of the tensile test samples were studied using the ZEISS scanning electron microscope previously mentioned. Because the samples used in this method must be conductive, the specimens underwent previous preparation, which included applying a layer of gold measuring 5 nanometers thick on the surface to be analyzed. This was performed with a SPI module metallizer (Sputter Coater model). A total of 18 samples was studied: a pair of 5%, 10%, 20%, and 30% fiber inclusion specimens; three pairs of 40% fiber inclusion specimens; two pairs of the 45% fiber inclusion specimens.

### 2.4. Mechanical Characterization

The mechanical characterization was carried out using tensile, bending, stepped creep, and stress relaxation tests, and an impact test. The tensile test was carried out under the ASTM D638-14 standard, under a constant load at a speed of 1 mm/min until the specimen was fractured, in a Universal Testing Machine WDW-2006. This test determined properties such as elongation up to failure, maximum tensile strength, modulus of elasticity, and load levels for subsequent staggered creep and relaxation tests. The bending test was carried out based on the ASTM D7264-15 standard on the same universal machine mentioned above at a 1 mm/min speed in a three-point configuration. The impact tests were conducted under the ISO 179-1 standard for plastic materials, using Gunt Hamburg WP 400 Pendulum Impact Tester equipment. The hammer was released at a height of 745 mm and adjusted to a weight of 2.05 kg to use a minimum energy scale of 15 J. The staggered creep test was performed at a speed of 5 mm/min, and they were applied at different load levels. For this test, constant loads were applied quickly at intervals of 1000 s to obtain the maximum deformations of the internal tensions of each probe and to analyze the viscoelastic behavior of the material. These loads consider the breaking load obtained in the tensile test as 100% and each level will correspond to a 10, 20, and 30% of this, respectively. The stress relaxation test was also performed at a speed of 5 mm/min, applying to the tensional state equivalent of 30% of the breaking load obtained in the tensile test. Once the tensional state is reached, this deformation is maintained for a time interval of 3000 s. It should be noted that the analysis was used in less than five samples for each test.

The denomination and composition of the specimens are presented in Table 2.

## 3. Results

### 3.1. Energy-Dispersive X-ray Spectroscopy (EDS)

**Figure 2:**
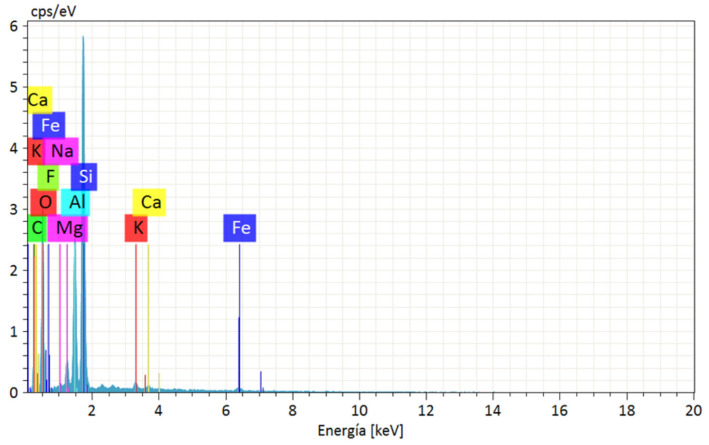
Bentonite EDS analysis.

The EDS analysis on the bentonite sample verified that the montmorillonite clay contains 0.40% calcium and 0.22% sodium, which allows it to be classified as calcium-type bentonite. In addition, large amounts of silicon and aluminum were found, 16.93% and 7.75%, respectively; this is the characteristic composition of smectic clays. This is presented in the diffractogram of Figure 2, where the peaks of appreciable intensity are indicated for the newly named elements. Table 3 presents the chemical composition of the sample and the amount of each component.

### 3.2. Scanning Electron Microscopy (SEM)

**Figure 3:**
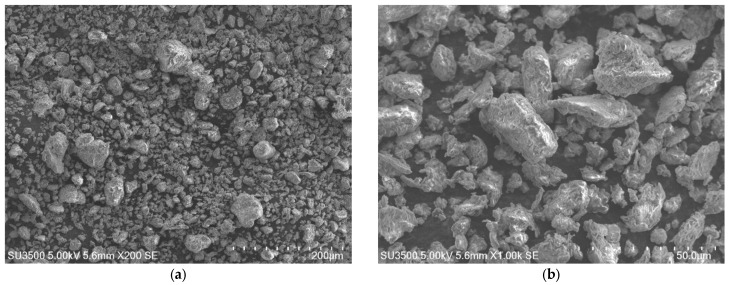
SEM analysis of bentonite samples: (**a**) grain size distribution, 200×; (**b**) grain surface irregularity, 1000×.

The results obtained by the SEM analysis of the untreated bentonite samples are shown in Figure 3. This identifies the characteristics of laminar-type smectic mineral with cavities between the discontinuous layers of its basic structural units and a rough surface suitable for interaction with the polyester resin. It can be observed that the size of the dispersed bentonite particles ranges from 2 µm to 60 µm in diameter, exposing the size variability that exists in the clay dimension. These characteristics, as has been presented by Fernández et al. [26] and Ollier et al. [27], favor the interface between polyester resin and bentonite. The variation in particle size guarantees a better interaction with the polymeric matrix, when small grains allow better results under tensile stress due to the increase in the particle surface, as presented by Onyedika et al. [28].

**Figure 4:**
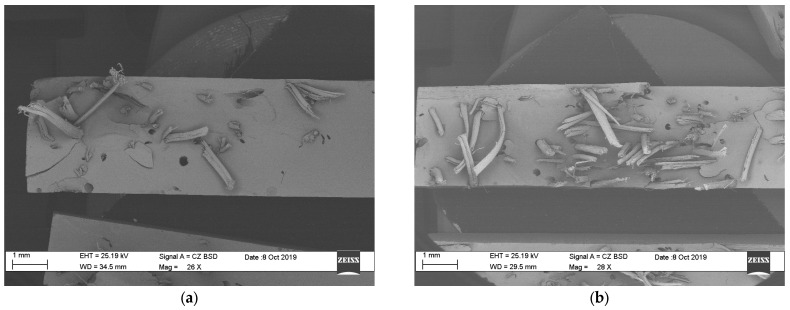

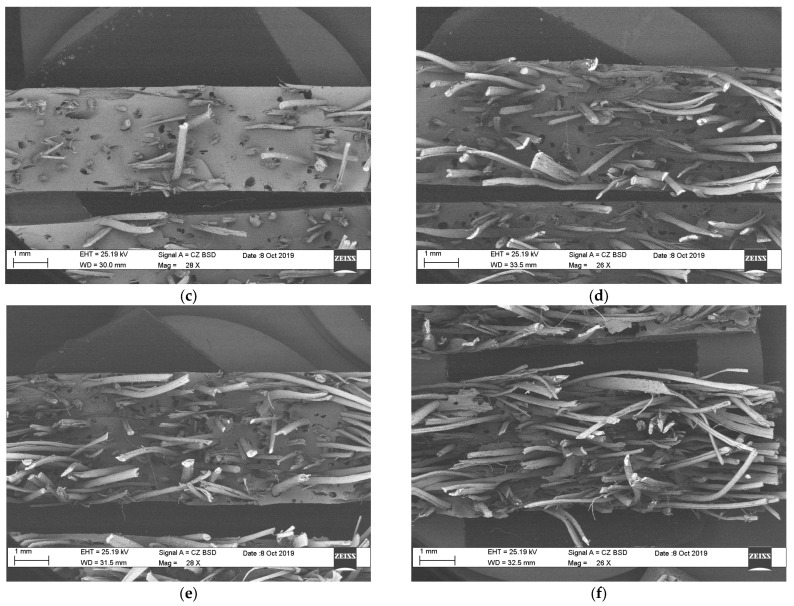
Distribution of fibers in the matrix: (**a**) F5, (**b**) F10, (**c**) F20, (**d**) F30, (**e**) F40, and (**f**) F45.

Figure 4 shows the distribution of fibers within the matrix as a function of the percentage of fiber addition. For specimens F5 and F10, Figure 4a,b, respectively, the distribution of the fibers is heterogeneous, with a greater number of them spread across more areas. Due to its distribution and the small number of fibers within the matrix, a small percentage of the stress is supported by the fibers, which generates a low influence on the mechanical properties of the composite material. For specimens F20, F30, F40, and F45, shown in Figure 4c–f, respectively, the homogeneity in the distribution increases in comparison with specimens F5 and F10, which translates into an improvement in the resistance to tension, as could be verified in the tensile test. This is because increasing fiber supports the tension exerted on the test samples.

**Figure 5:**
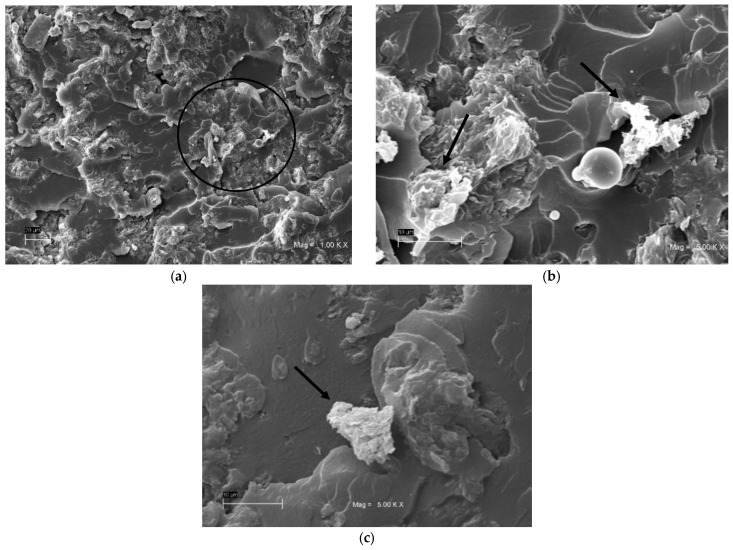
Bentonite in the matrix, BF5 sample: (**a**) 1000×; (**b**) zoomed, with the circle zone at 5000×—note the variability of sizes and the morphology of bentonite particles; (**c**) microscopy at 5000×, loose particle.

The materials used as reinforcement, i.e., the long sisal fibers and the bentonite particles, were used without chemical, mechanical, or other surface treatments that would modify their interaction in the union or interface, with the polymer used as the matrix. Due to the hydrophilic nature of both vegetable fibers and clay and the hydrophobic nature of polyester, neither chemical nor diffusion bonds were established between them, with the union being purely mechanical [29]. Due to the above reasons, the transfer of the stresses assumed by the fibers is conditioned by their length/diameter ratio and length, making it necessary to point out that the one used is greater than the critical length of 50 mm. Similarly, the bentonite particles establish a mechanical bond with the matrix and restrict its deformations in its vicinity, slightly increasing its rigidity and tensile strength. This means that it becomes convenient to use particles with the smallest possible size, which therefore have as large an effective area as possible. The mechanical union is influenced by the topography of these elements, with the roughness appreciated in the fibers and the beneficial particles for said union (see Figure 4 and Figure 5). Although the treatments above allow for increasing the “strength of the union in the interfaces” and influencing the compound’s average global effective properties, these were not evaluated in the present investigation; we recommend they are studied in further investigations.

Figure 5 shows the surface of the composite with 5% fiber inclusion BF5, where details of the bentonite particles can be seen, showing heterogeneity in their size and shapes, as observed in the delimited area of Figure 5a. The figure also shows the formation of agglomerates and the existence of loose particles not adhered to the matrix, as observed in the points marked by arrows in Figure 5b and the point indicated in Figure 5c, respectively. This suggests that the mixing procedure was not effective enough to homogeneously disperse the nanoclay in the polymer matrix, also reflecting a low level of interaction between the matrix and the reinforcement. These agglomerations have been observed in previous investigations, such as the one carried out by Jastrzebska et al. [30]; they mean that there is a problem in the decrease in the mechanical properties of the composite material as there is an incomplete dispersion of the particles in the polyester matrix. This can generate undesired stress concentrators, and, in the first instance, this defect may be because the size of the bentonite is not entirely adequate as it presents a variety of grain sizes, as can be verified through the use of scanning electron microscopy.

**Figure 6:**
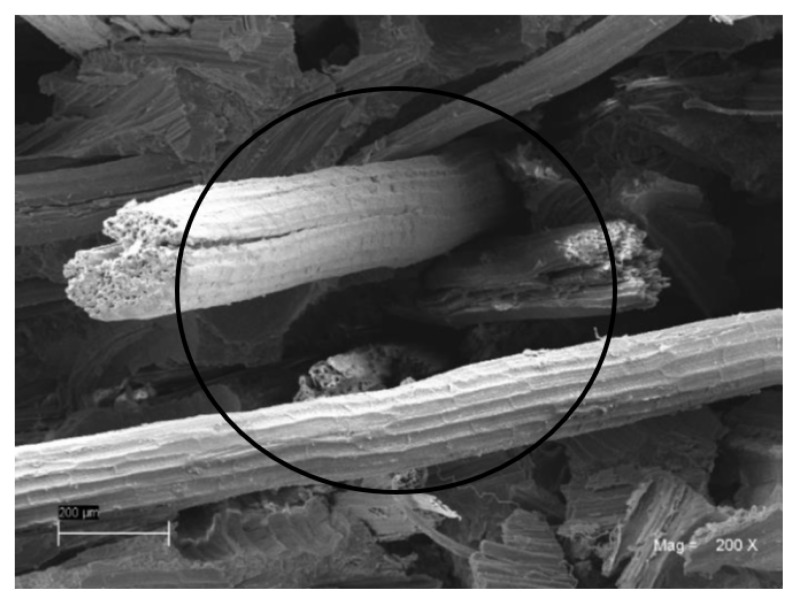
Absence of resin between several fibers together in the sample BF45, microscopy at 200×.

Likewise, defects in the matrix–reinforcement interface were found in the samples with 45% addition of fibers, FB45. One of these are the areas with high fiber density, as seen in the demarcated area of Figure 6, which has areas with an absence of resin and several fibers together at its base. This explains the decrease in mechanical properties in the tensile test for the BF45 specimen.

### 3.3. Tensile Test

**Figure 7:**
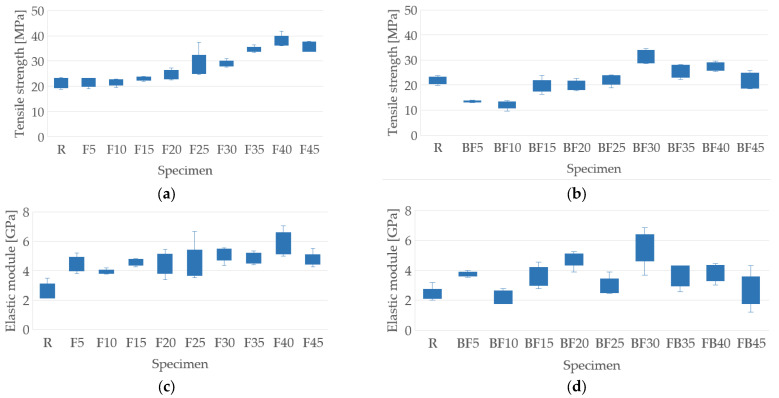
Tensile test results: (**a**) tensile strength of samples with the addition of fibers only; (**b**) tensile strength of samples with the addition of fibers and bentonite; (**c**) elastic module of samples with the addition of fibers only; (**d**) elastic module of samples with the addition of fibers and bentonite.

The results of the tensile test are presented in Figure 7. It can be seen in Figure 7a that the samples that do not contain bentonite show an increase in tensile strength compared to the resin samples, where the maximum value of 38.01 MPa was obtained for the F40 samples, representing an increment of 43.09%. On the other hand, the samples that contain bentonite also present an increase in resistance, but this is perceived from a 25% fiber addition, where the maximum value of 31.39 MPa was obtained for the BF30 samples; this represents an increment of 31.09% concerning to the resin samples. This behavior is a result of the fact that fiber density within the matrix is sufficient in supporting a uniform stress state, as has been presented by Guerra-Silva et al. [11], Soto et al. [15], and Fernandez et al. [26]. The addition of bentonite does not significantly influence the tensile strength, since only the BF30 sample presented an increase in resistance of 2.45% concerning its F30 pair. Similar behavior was observed by Fernández et al. [26] and this was seen to be due to the influence of bentonite agglomerations within the polyester matrix, which function as stress concentrators.

The elastic modulus for the samples that do not contain bentonite increases as the percentage of fiber addition increases, with a maximum value of 5.83 GPa for the F40 samples, representing an increase of 32.93% in the resin samples, as can be seen in Figure 7c. The preceding is influenced by the increase in the resistance and the limit deformation of the composite, as well as the modification of the mechanical behavior of the matrix in the vicinity of the interface with the fibers, increasing the overall effective stiffness of the composite material, as also discussed by Soto et al. [15] and Fernandez et al. [26]. For the samples containing bentonite, the elastic modulus increases for all the percentages of fiber addition except for the BF10 samples, which show a decrease of 16.22% in the resin samples. This may be due to a defect caused during the manufacturing process, such as the resin not being distributed evenly among the fibers, or the bentonite particles forming agglomerates, creating stress concentrations. The maximum value reached was 5.51 GPa for the BF30 samples, representing an increase of 53.18% for the resin samples, as seen in Figure 7d. The presence of bentonite in the samples does not improve the elastic tensile modulus; of all the specimens, only BF20 and BF30 presented increases of 6.26% and 8.04% for their pairs, F20 and F30, respectively. This behavior may be due to the percentage of bentonite used; for example, Fernández et al. [26] obtained increases for all specimens studied except one, which used a 5% addition of bentonite. A summary of the results is presented in Table 4. The Supplementary Materials (Figures S1 and S2) include additional data, such as the stress vs. strain curves.

### 3.4. Flexural Test

**Figure 8:**
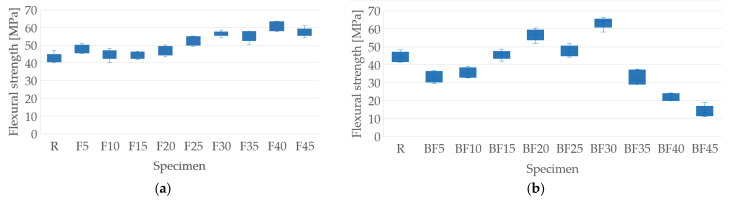

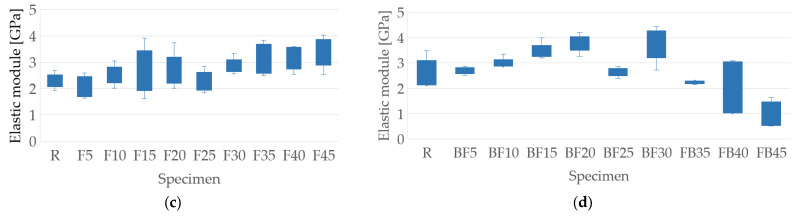
Flexural test results: (**a**) flexural strength of samples with the addition of fibers only; (**b**) flexural strength of samples with the addition of fibers and bentonite; (**c**) elastic module of samples with the addition of fibers only; (**d**) elastic module of samples with the addition of fibers and bentonite.

The results shown in Figure 8 were obtained from the bending tests. Figure 8a,b show the flexural strength results for the samples containing only fibers and those containing fibers and bentonite, respectively. In contrast, Figure 8c,d present the flexural elastic modulus for the samples with fibers without and with the addition of bentonite, respectively.

Figure 8a shows that flexural resistance increases as the percentage of fiber addition increases, compared to the values obtained for the resin samples. This may be due to the contribution of fiber stiffness to the matrix; this is in agreement with the results obtained by Fernández et al. [26]. The maximum value obtained was 60 MPa for the F40 samples, representing an increase of 29.93% compared to the resin samples. Once the bentonite is added, the flexural strength is affected; this is because its value decreases for more of the samples compared to the resin samples. The maximum value found was 63.49 MPa, representing an increase of 32.68% compared to the samples with resin. The addition of bentonite in the samples does not significantly improve the flexural strength, since only three specimens showed improvements compared to those without bentonite, as seen in Figure 8a,b. This is the case of BF 15, BF20, and BF30, whose resistance values of 45.71, 56.62, and 63.49 MPa represent increases of 2.72, 21.01, and 12.39% for their F15, F20, and F30 pairs, respectively. This increase and then decrease in resistance may be due to the percentage of bentonite used (7%), since a progressive increase in property has been perceived for 5%, as reported by Fernández et al. [26].

The elastic modulus, on the other hand, increases as the percentage of fibers in the matrix is higher, as can be seen in Figure 8c. The maximum value obtained was 3.35 GPa for the F45 specimen, representing an increase of 31.34% compared to the one that only contains resin. The samples with bentonite show an increase in their elastic moduli up to a 20% addition of fibers, which then decrease after a 30% addition of fibers. The BF20 specimen was the one that presented the best results, with a value of 3.78 MPa, which represents an increase of 39.15% for the resin specimen. Unlike the resistance, the elastic modulus do present significant improvements once the bentonite is added compared to the samples that do not contain it. This is evidenced for the specimens BF5, BF10, BF15, BF20, BF25, and BF30, which presented increases of 32.20, 19.12, 29.21, 42.64, 16.30, and 30.88% with respect to their pairs, F5, F10, F15, F20, F25, and F30, respectively. Compared with the results obtained by Fernández et al. [26], it can be deduced that 7% bentonite further favors the formation of agglomerates in the composite material, which means that, from a particular value of fiber addition (30%), the elastic modulus begins to decrease. A summary of the results is presented in Table 5. Additional data for the stress vs. strain curves are included in the Supplementary Materials (Figures S3 and S4).

### 3.5. Step Creep Test

For the stepped flow test, five specimens were tested for each percentage of fiber inclusion. The specimens with the best results are presented in Table 6. For each load step, the composite material presented an immediate elastic deformation component (Ɛe) and a viscous deformation component (Ɛv), with the latter increasing over time.

The maximum viscoelastic deformations were reached in the material with 35% fiber inclusion with a value of 0.58%, making it clear that, from the percentage, the material can no longer maintain constant stresses without drastically deforming. The maximum value of the total deformation was also obtained for BF35 with a value of 1.34%, being 309% and 236% more than those presented by FB25 and FB30, respectively. These results agree with those presented by Poilane et al. [31], where the residual deformations occur at levels above the elastic limit; this can be attributed to the mechanisms of adhesion and sliding occurring within the fibers in the fiber wall [32].

**Figure 9:**
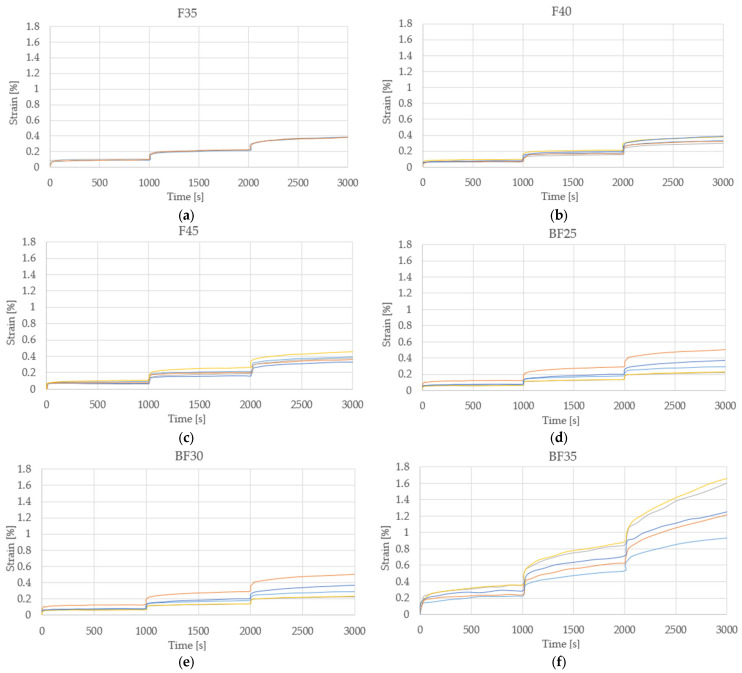
Step creep test with color-differentiated samples: (**a**) F35, (**b**) F40, (**c**) F45, (**d**) BF25, (**e**) BF30, and (**f**) BF35.

Figure 9 shows the graphs that show the behavior of the tested specimens, where each colored line represents the behavior of each of the samples. Through the analysis of these, it is perceived that, as the percentage of fiber addition increases, the dispersion between trials of the same percentage increases. The specimen that showed the highest values of elastic and volumetric deformation is BF35, as can be seen in Figure 9f; in turn, this is the one that presented the most dispersion compared to the rest. This is due to the presence of bentonite, since the samples that only contain fiber and that have a lower percentage of it exhibited more homogeneous behavior.

### 3.6. Relaxation Essay Test

**Figure 10:**
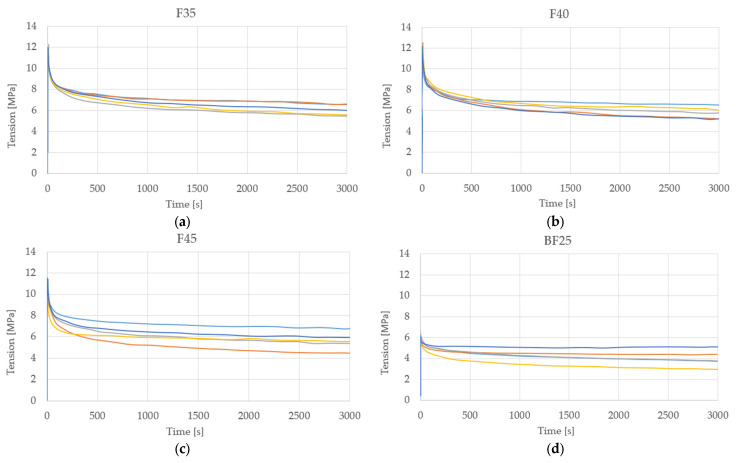

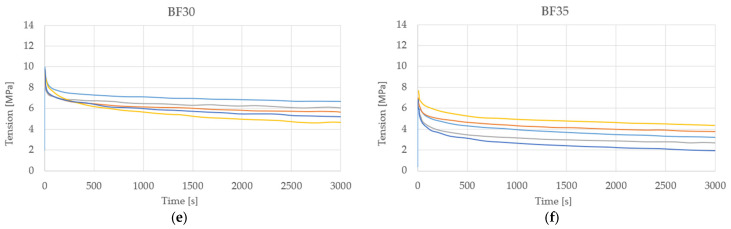
Relaxation essay test, color-differentiated samples: (**a**) F35, (**b**) F40, (**c**) F45, (**d**) BF25, (**e**) BF30, and (**f**) BF35.

The optimal results from the relaxation test are presented in Table 7. The specimen that presented the best relaxation stress was F35 with a value of 6.04 MPa, which, compared to its pair BF35, shows an increase of 46.5%. These results are similar to those obtained by Poilane et al. [31] and Sala et al. [33]. A stiffening effect was perceived during the recovery phase and the relaxation stress is affected by the bentonite particles, which may be associated with a dependence on the material’s stiffness. Figure 10 shows the graphs that show the behavior of the tested specimens, where each colored line represents the behavior of each of the samples.

### 3.7. Impact Test

**Figure 11:**
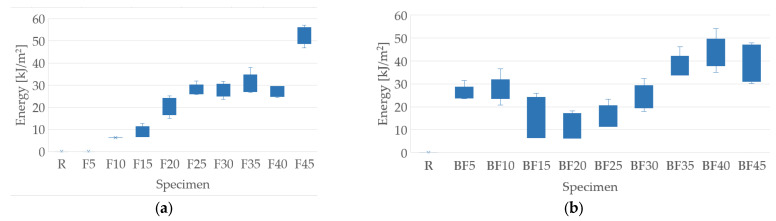
Impact test results: (**a**) samples with the addition of fibers only; (**b**) samples with the addition of fibers and bentonite.

The results obtained from the impact test carried out are shown in Figure 11. Due to the scale used by the team, the R and F5 specimens did not register adsorption in any of the test tubes, as can be seen in Figure 11a. In the specimens that do not contain bentonite, an increase in the absorption of impact energy was observed from specimen F15 to specimen F45, with F45’s maximum value being 52.41 kJ/m^2^. Once the bentonite was added, an increase in the energy absorbed was observed, with a subsequent decrease in its properties. The BF40 specimen was the one that presented the highest energy absorption, with a value of 43.86 kJ/m^2^, as shown in Figure 11b. Bentonite improves the impact resistance of the composite material, which is evidenced by comparing the specimens F10, F15, F35, and F40 with their pairs BF10, BF15, BF35, and BF45, whose increase values were 21.28, 48.85, 22.0, and 61.61%, respectively. These results are similar to those obtained by Fernández et al. [34] and Suriyaprakash et al. [35]. The decrease in the impact resistance between the pairs containing 20, 25, and 30% fiber inclusion could be due to the formation of agglomerates, caused by the presence of bentonite, which translates into stress concentrations in the composite material matrix.

## 4. Conclusions

Polyester hybrid composites were fabricated with sisal fiber in percentages between 5 and 45%, with a fixed percentage of bentonite at 7%. For all the manufactured specimens, a good distribution of fibers was achieved, as well as a good adhesion between the fibers, the bentonite, and the matrix.

A percentage of 7% bentonite does not improve the tensile strength of the composite material, except for the specimen with 30% addition of fiber, which presented an increment of 33.8% in relation to its pair. The tensile elastic modulus is affected by this, presenting a decrease in its value compared to the specimens that do not contain bentonite, except for the 20% and 30% addition of fiber, the increment values of which were 6.3% and 8% compared to their peers, respectively.

The presence of bentonite favored the flexural strength with increases of 2.7, 21, and 12.4% in the specimens with 15, 20, and 30% addition of sisal, respectively. At the same time, the elastic modulus presents a significant improvement for almost all of the specimens compared to their peers that do not contain bentonite, with a maximum increase of 43% for a 20% addition of fiber.

The viscoelastic behavior of the material becomes more evident after a 25% addition of sisal fiber and improves significantly when bentonite is added to the matrix. The maximum viscoelastic deformation of 0.58% was reached in samples with a 35% addition of fibers. The relaxation stress is not favored with the addition of bentonite; this value decreased substantially with the presence of bentonite, possibly due to its interaction with sisal fibers.

Impact resistance improved considerably for almost all of the specimens studied; this led to the deduction that bentonite is favorable in the composite material. The most significant increase found was 62%, shown in the sample with a 40% addition of the fiber.

## Figures and Tables

**Table 1:** Physics properties of POLIPOL 3401-H15 polyester.

Properties	Typical Values
(Density, g/cm^3^)	1.128
Refractive index	1.547
Acidity index (mg KOH/g)	24
Brookfield^®^ viscosity (cp)	550
Thixotropy	N/A
Gel time (min)	11
Monomer content (%)	39
Flashpoint (°C)	33
Degradation temperature (°C)	170

**Table 2:** Specimens’ composition.

Specimen	Polyester Resin (R) [%]	Sisal Fiber (F) [%]	Bentonite (B) [%]
R	100	-	-
F5	95	5	-
F10	90	10	-
F15	85	15	-
F20	80	20	-
F25	75	25	-
F30	70	30	-
F35	65	35	-
F40	60	40	-
F45	55	45	-
BF5	88	5	7
BF10	83	10	7
BF15	78	15	7
BF20	73	20	7
BF25	68	25	7
BF30	63	30	7
BF35	58	35	7
BF40	53	40	7
BF45	48	45	7

**Table 3:** Bentonite chemical compositions.

Element	At. No.	Netto	Mass [%]	Mass Norm. [%]	Atom [%]	Abs. Error [%] (1 Sigma)	Real. Error [%] (1 Sigma)
Oxygen	8	5765	40.43	42.30	43.47	6.20	15.33
Carbon	6	1635	27.60	28.93	39.61	5.53	19.88
Silicon	14	15,592	16.18	16.93	9.91	0.74	4.56
Aluminum	13	5906	7.41	7.75	4.72	0.41	5.49
Magnesium	12	930	1.41	1.48	1.00	0.13	9.07
Iron	26	278	0.85	0.88	0.26	0.08	9.92
Fluorine	9	59	0.52	0.55	0.47	0.35	66.87
Potassium	19	353	0.52	0.54	0.23	0.06	11.51
Calcium	20	228	0.38	0.40	0.16	0.05	14.35
Sodium	11	94	0.22	0.23	0.16	0.06	26.31
		Sum	95.57	100.00	100.00		

**Table 4:** Tensile test results summary.

Specimen	Tensile Strength (MPa)	Increase/ Decrease	Elastic Module (GPa)	Increase/ Decrease
R	21.63	-	2.58	-
F5	21.59	-	4.41	-
BF5	13.54	−37.29	3.76	−14.74
F10	21.54	-	3.93	-
BF10	12.06	−44.01	2.22	−43.51
F15	23.01	-	4.61	-
BF15	19.45	−15.47	3.61	−21.69
F20	24.52	-	4.47	-
BF20	20.00	−18.43	4.75	6.26
F25	26.23	-	4.40	-
BF25	22.13	−15.63	2.93	−33.41
F30	28.94	-	5.10	-
BF30	31.39	33.72	5.51	8.04
F35	34.53	-	4.81	-
BF35	25.41	−26.41	3.67	−23.70
F40	38.01	-	5.83	-
BF40	27.37	−27.99	3.81	−34.65
F45	35.89	-	4.74	-
BF45	21.99	−38.73	2.64	−44.30

**Table 5:** Flexural test results summary.

Specimen	Flexural Strength (MPa)	Increase/ Decrease	Elastic Module (GPa)	Increase/ Decrease
R	42.74	-	2.3	-
F5	47.8	-	2.05	-
BF5	32.99	−30.98	2.71	32.20
F10	44.98	-	2.51	-
BF10	35.58	−20.90	2.99	19.12
F15	44.5	-	2.67	-
BF15	45.71	2.72	3.45	29.21
F20	46.79	-	2.65	-
BF20	56.62	21.01	3.78	42.64
F25	52.62	-	2.27	-
BF25	47.63	−9.48	2.64	16.30
F30	56.49	-	2.85	-
BF30	63.49	12.39	3.73	30.88
F35	55.19	-	3.07	-
BF35	32.83	−40.51	2.23	−27.36
F40	61	-	3.14	-
BF40	21.8	−64.26	1.93	−38.54
F45	57.24	-	3.35	-
BF45	14	−75.54	0.61	−81.79

**Table 6:** Elastic and viscoelastic deformations.

Specimen	Step 1		Step 2		Step 3	
	Ɛ_e_ * (%)	Ɛ_v_ ** (%)	Ɛ_e_ (%)	Ɛ_v_ (%)	Ɛ_e_ (%)	Ɛ_v_ (%)
F35	0.06	0.03	0.08	0.04	0.06	0.10
F40	0.06	0.02	0.06	0.05	0.07	0.08
F45	0.07	0.02	0.07	0.06	0.06	0.10
BF25	0.06	0.02	0.06	0.05	0.06	0.07
BF30	0.06	0.03	0.07	0.07	0.06	0.11
BF35	0.12	0.18	0.10	0.32	0.04	0.58

Ɛ_e_ *—elastic deformation; Ɛ_v_ **—viscoelastic deformation.

**Table 7:** Relaxation test results.

Specimen	Maximum Tension (MPa)	Relaxation Tension (MPa)	Relaxation Module (GPa)
F35	11.72	6.04	2.24
F40	12.2	5.75	2.15
F45	11.17	5.61	2.06
BF25	6.41	4.01	2.64
BF30	9.07	5.62	3.9
BF35	7.23	3.23	1.55

## Data Availability

The data presented in this study are available on request from the corresponding author.

## Supplementary Materials

The following supporting information can be downloaded at: https://www.mdpi.com/article/10.3390/polym15193963/s1. Figure S1: Tensile strength results of samples with the addition of fibers only: (a) R; (b) F5; (c) F10; (d) F15; (e) F20; (f) F25; (g) F30; (h) F35; (i) F40; and (j) F45.; Figure S2: Tensile strength results of samples with the addition of fibers and bentonite: (a) R; (b) BF5; (c) BF10; (d) BF15; (e) BF20; (f) BF25; (g) BF30; (h) BF35; (i) BF40; and (j) BF45; Figure S3: Flexural strength results of samples with the addition of fibers only: (a) R; (b) F5; (c) F10; (d) F15; (e) F20; (f) F25; (g) F30; (h) F35; (i) F40; and (j) F45; Figure S4: Flexural strength results of samples with the addition of fibers and bentonite: (a) R; (b) BF5; (c) BF10; (d) BF15; (e) BF20; (f) BF25; (g) BF30; (h) BF35; (i) BF40; and (j) BF45.
