# Supplementary material for: Influence of Bentonite Particles on the Mechanical Properties of Polyester–Sisal Fiber Composites
Source: Polymers (Basel). 2023 Sep 30;15(19):3963. doi: 10.3390/polym15193963 (PMC10575223; doi:10.3390/polym15193963)
Supplement: Supplementary file 1 [file polymers-15-03963-s001.zip › polymers-2593692-supplementary.pdf]

Figure S1 presents the graphs with the stress vs strain curves of the tensile test for the specimens that only contain fiber addition.

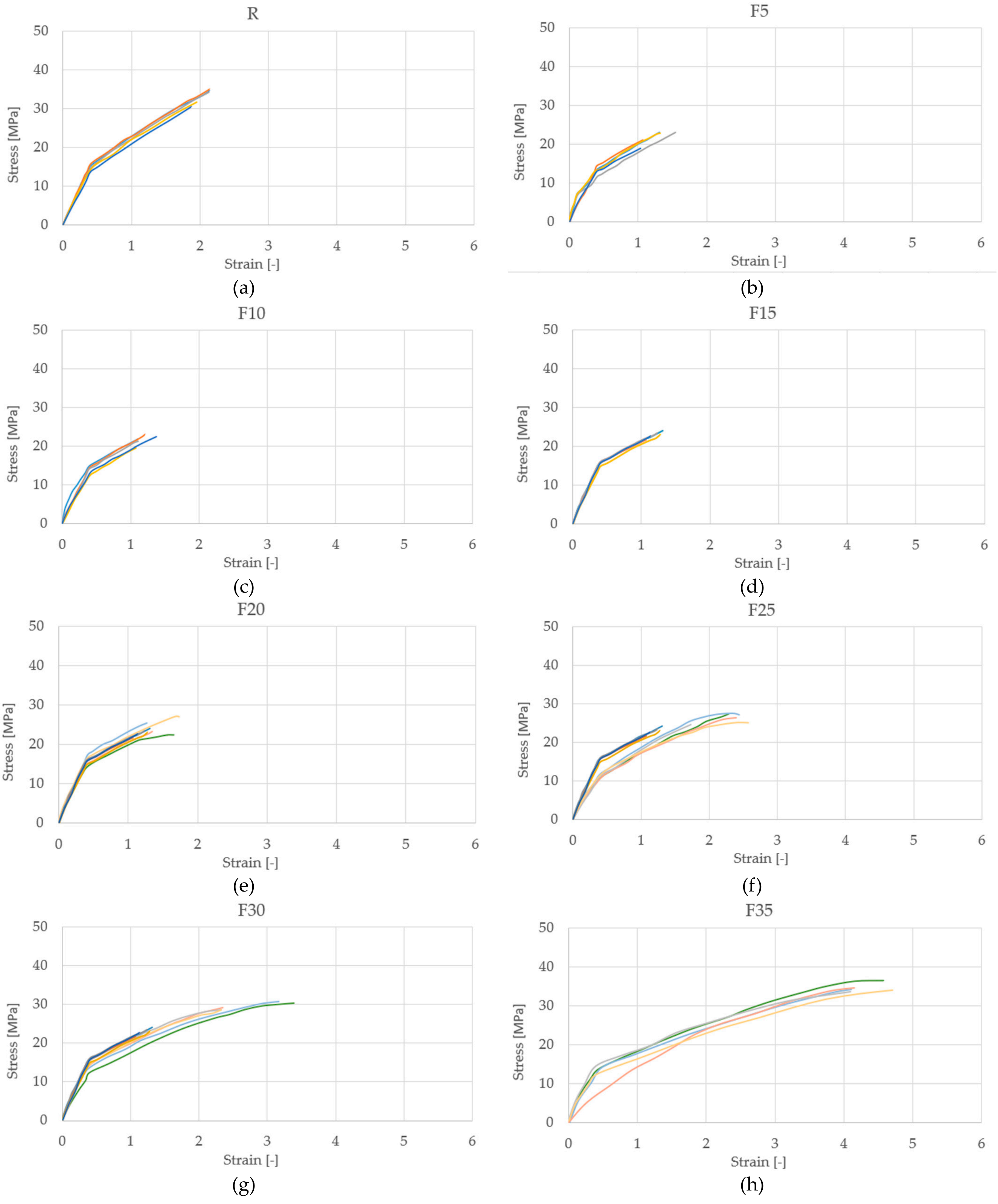

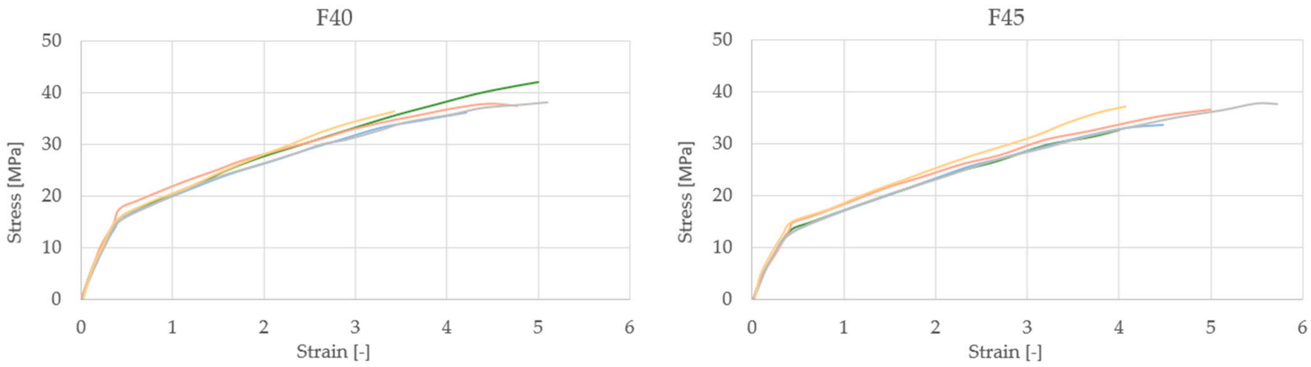

(i) (j)

Figure S1. Tensile strength results of samples with the addition of fibers only: (a) R; (b) F5; (c) F10; (d) F15; (e) F20; (f) F25; (g) F30; (h) F35; (i) F40; and (j) F45.

Figure S2 presents the graphs with the stress vs strain curves of the tensile test for the specimens containing fiber and bentonite addition.

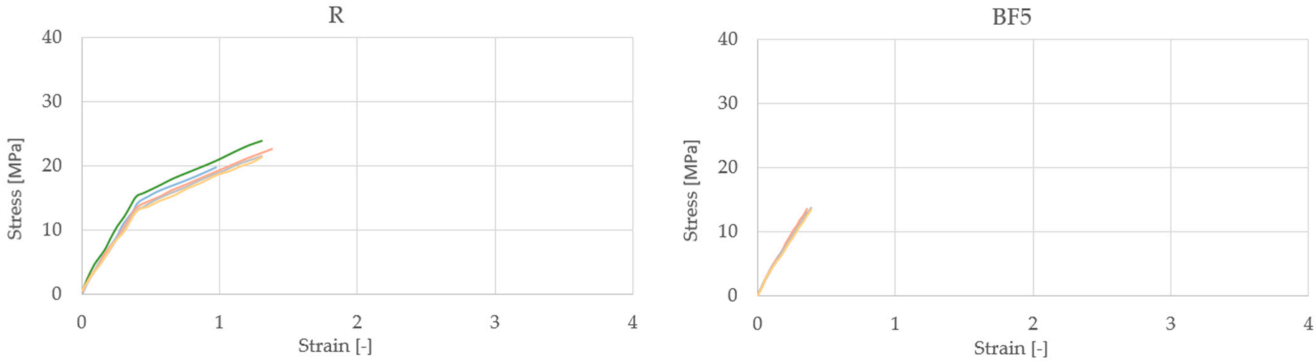

(a) (b)

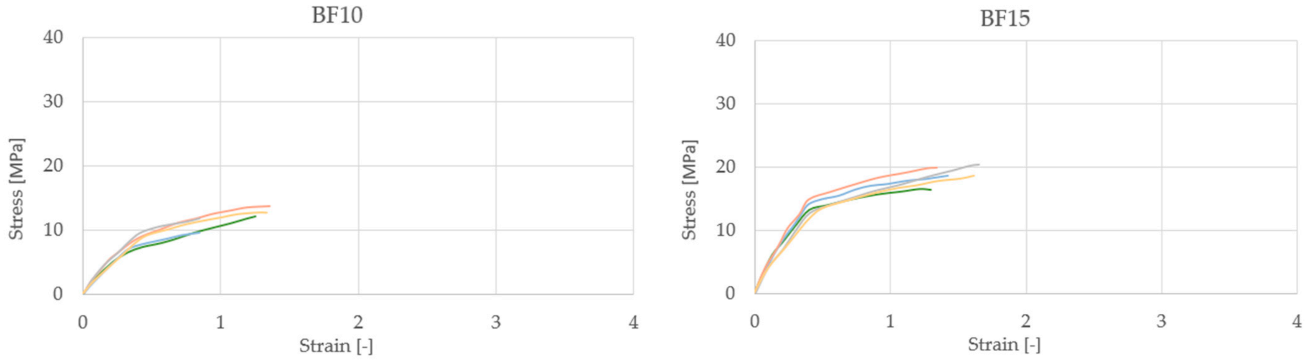

(c) (d)

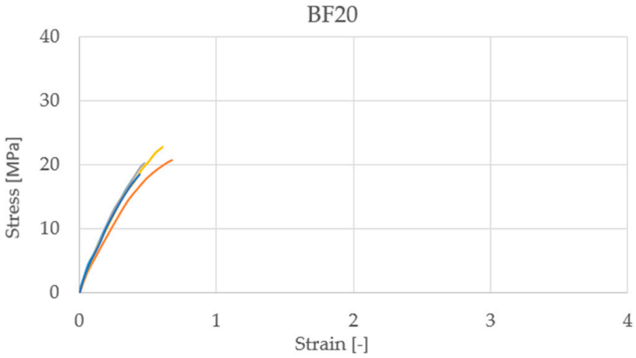

(e)

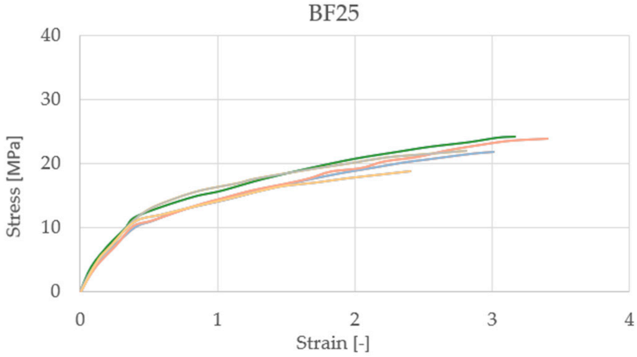

(f)

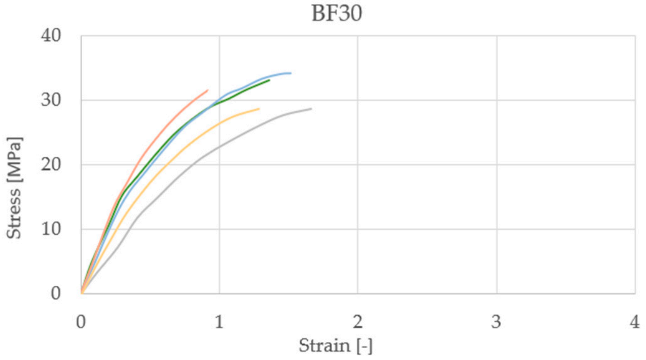

(g)

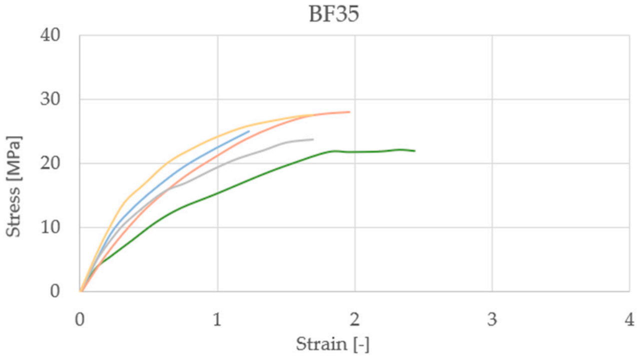

(h)

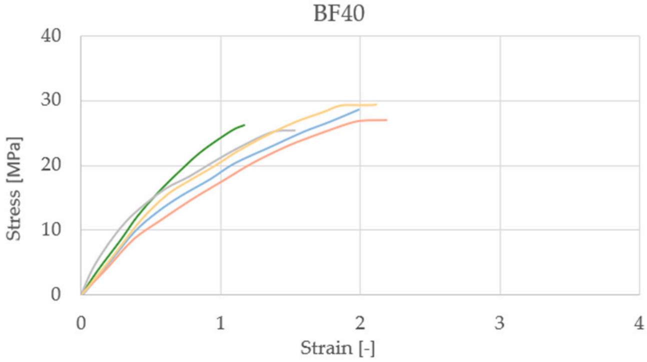

(i)

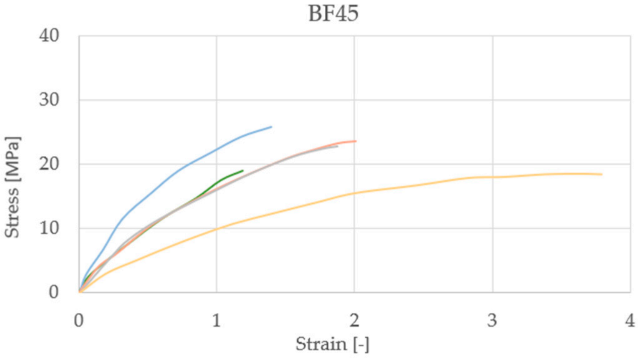

(j)

Figure S2. Tensile strength results of samples with the addition of fibers and bentonite: (a) R; (b) BF5; (c) BF10; (d) BF15; (e) BF20; (f) BF25; (g) BF30; (h) BF35; (i) BF40; and (j) BF45.

Figure S3 presents the graphs with the stress vs strain curves of the flexural test for the specimens that only contain fiber addition.

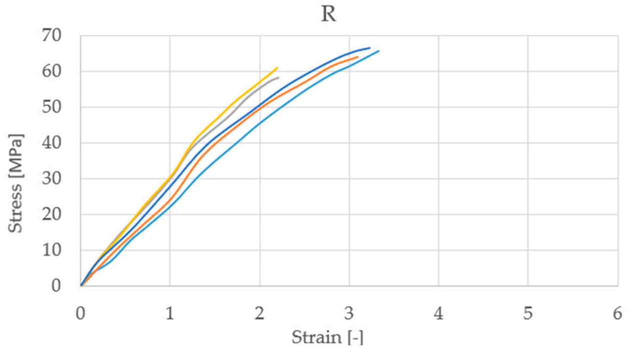
R
Stress [MPa]
Strain [-]

(a)

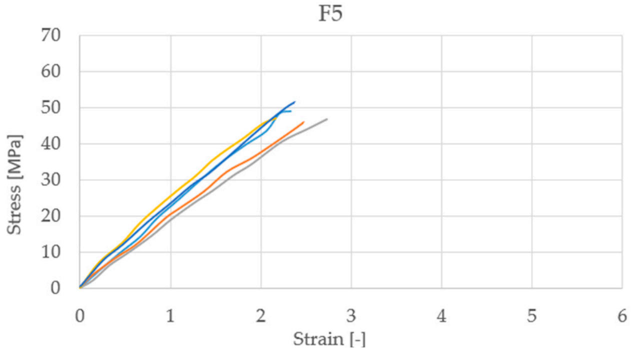
F5
Stress [MPa]
Strain [-]

(b)

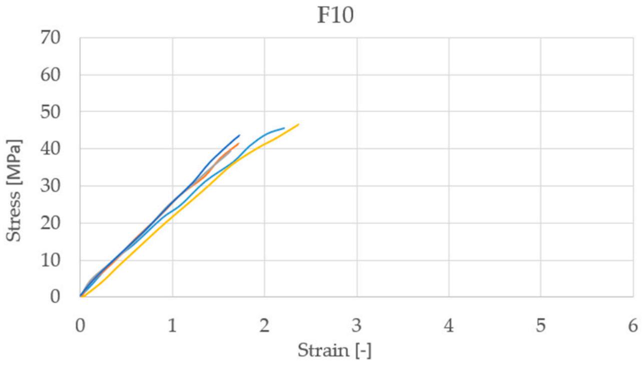
F10
Stress [MPa]
Strain [-]

(c)

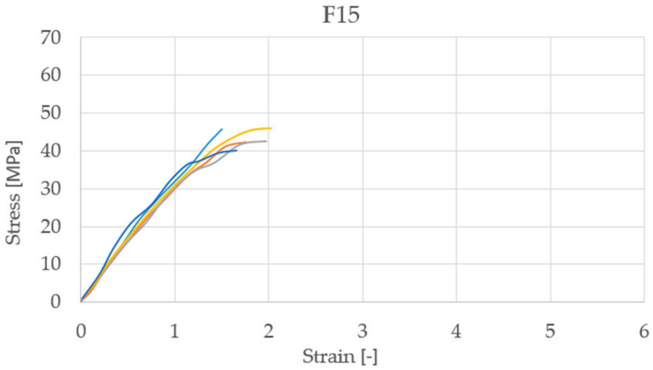
F15
Stress [MPa]
Strain [-]

(d)

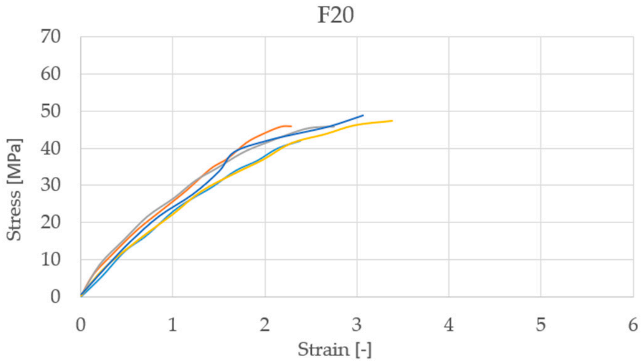
F20
Stress [MPa]
Strain [-]

(e)

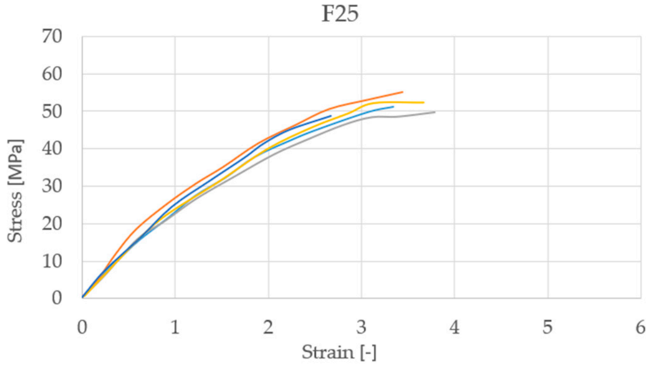
F25
Stress [MPa]
Strain [-]

(f)

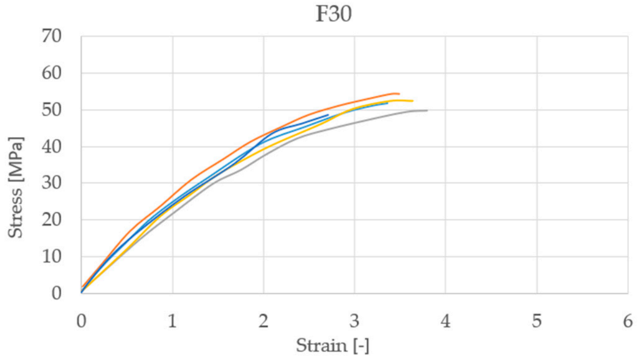
F30
Stress [MPa]
Strain [-]

(g)

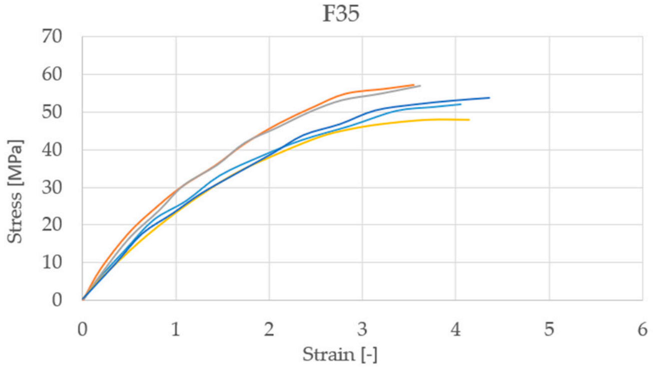
F35
Stress [MPa]
Strain [-]

(h)

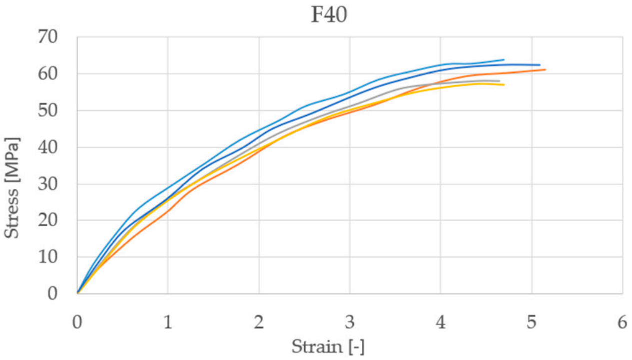

(i)

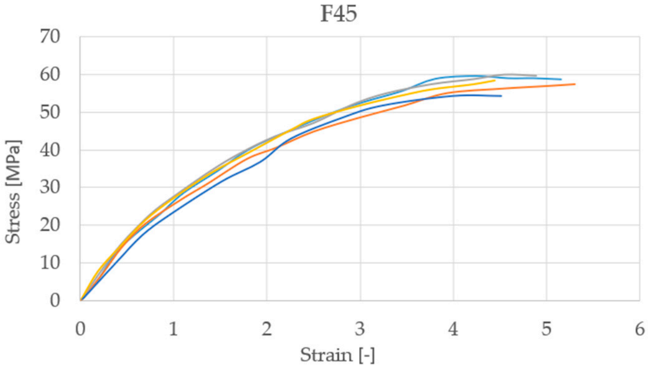

(j)

Figure S3. Flexural strength results of samples with the addition of fibers only: (a) R; (b) F5; (c) F10; (d) F15; (e) F20; (f) F25; (g) F30; (h) F35; (i) F40; and (j) F45.

Figure S4 presents the graphs with the stress vs strain curves of the flexural test for the specimens containing fiber and bentonite addition.

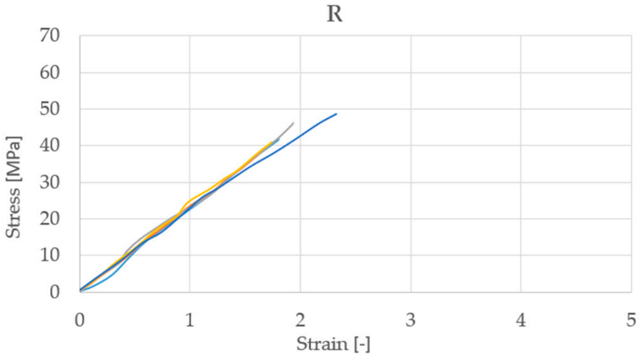

(a)

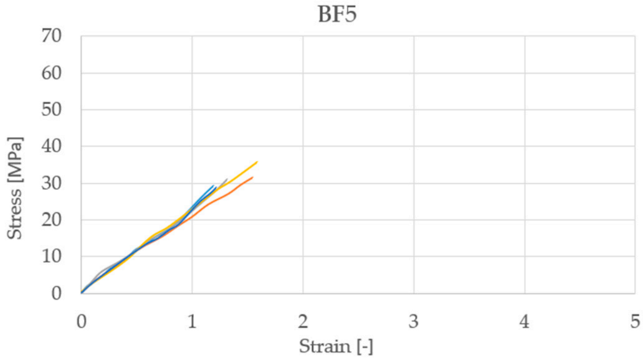

(b)

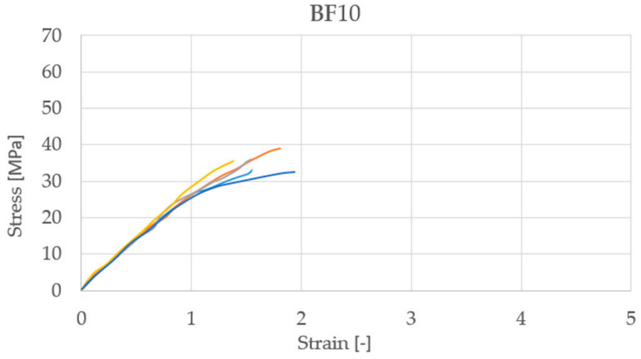

(c)

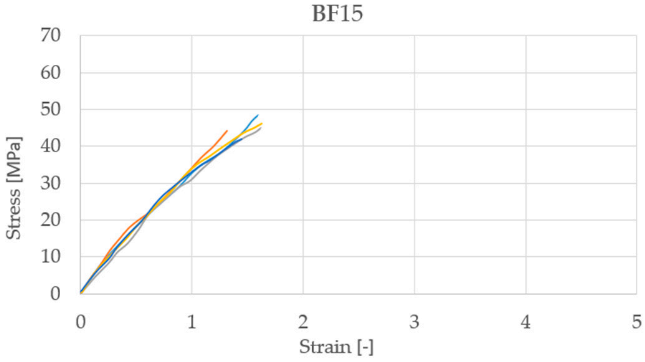

(d)

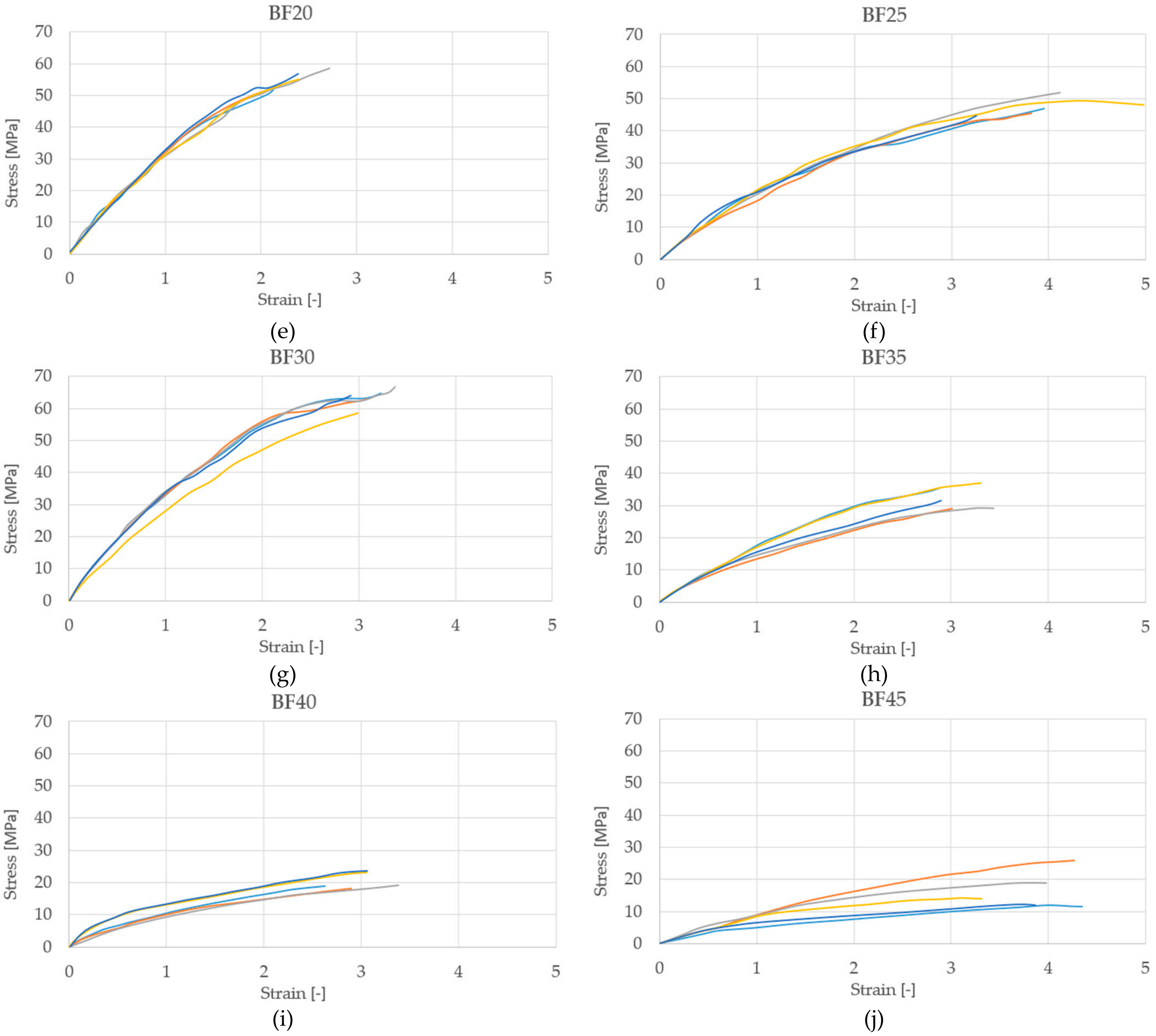

Figure S4. Flexural strength results of samples with the addition of fibers and bentonite: (a) R; (b) BF5; (c) BF10; (d) BF15; (e) BF20; (f) BF25; (g) BF30; (h) BF35; (i) BF40; and (j) BF45.
